# NRF2 activation in cancer cells suppresses immune infiltration into the tumor microenvironment

**DOI:** 10.1016/j.isci.2025.113519

**Published:** 2025-09-06

**Authors:** Huaichun Wen, Takafumi Suzuki, Anqi Zhang, Miu Sato, Mahiro Matsumoto, Yuka Takahashi, Yushi Takahashi, Masayuki Yamamoto

**Affiliations:** 1 Department of Biochemistry and Molecular Biology, Tohoku Medical Megabank Organization, Tohoku University, 2-1 Seiryo-machi, Aoba-ku, Sendai, Japan; 2 Advanced Research Center for Innovations in Next-Generation Medicine (INGEM), Tohoku University, Sendai 980-8575, Japan

**Keywords:** Cancer, Immunology, Molecular biology

## Abstract

Clinical observations have revealed that NRF2 hyperactivation in cancer cells is often associated with immune suppression in the tumor microenvironment. However, it remains unclear whether NRF2 hyperactivation directly reduces immune cell infiltration into tumors. To address this question, we established a syngeneic mouse model using the transplantation of 3LL lung cancer-derived cells with either NRF2 hyperactivation via *Keap1* gene deletion or concomitant *Keap1-Nrf2* gene deletion. A series of flow cytometry, histological analysis, and comprehensive gene expression profiling demonstrated that immune cell infiltration was significantly reduced in KEAP1-deleted tumors, with a marked decrease in CD45-positive cells, particularly myeloid and monocytic populations. In contrast, concomitant deletion of NRF2 restored immune cell infiltration in the KEAP1-deleted tumors. These findings provide convincing lines of evidence that NRF2 activation in cancer cells suppresses immune cell infiltration into tumors. Our study sheds light on the mechanistic basis by which NRF2 activation contributes to cancer malignancy.

## Highlights

• Syngeneic murine cancer models with NRF2-activation/repression are established
• NRF2 activation in cancers evokes low-level immune cell infiltration into tumors
• NRF2-activated cancers grow without inducing immune cells in systemic circulation

## Graphical abstract

**Figure:**
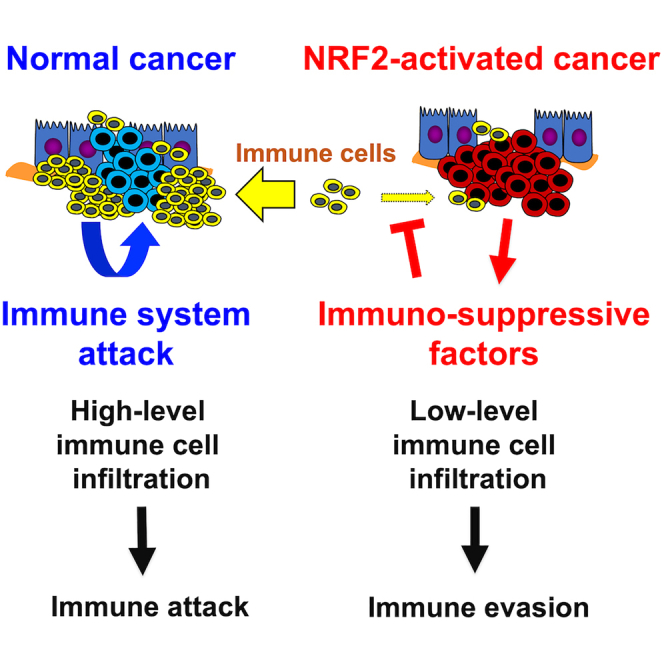

## Introduction

Lung cancer is the leading cause of cancer-related deaths worldwide.^1^ Especially, non-small cell lung cancer (NSCLC), including lung adenocarcinoma (LUAD) and squamous cell lung carcinoma, is a major part of lung cancer cases. Despite promising results of immune check-point blockade (ICB) agents for NSCLC treatments, either alone or simultaneously with chemo/radiotherapy, improvements in survival are still modest. Only a subset of patients achieves long-lasting remission.^2^ It has been shown that somatic mutations of NRF2 (Nuclear factor erythroid 2-related factor 2) and KEAP1 (Kelch-like ECH-associated protein 1) are observed at high frequency in NSCLC.^3^^,^^4^ NRF2 is a transcription factor that governs the expression of various cytoprotective genes in response to oxidative and electrophilic stresses.^5^^,^^6^^,^^7^ NRF2 activity is mainly regulated by KEAP1.^8^^,^^9^ Upon exposure to the stressors, KEAP1 reduces its ability to degrade NRF2,^10^ and consequently NRF2 accumulates and activates a set of cytoprotective genes.

For past two decades, it has been unveiled that NRF2 activity is hyperactivated in many types of cancers.^3^^,^^11^^,^^12^^,^^13^ These NRF2 hyperactivations are caused not only by somatic mutations in *KEAP1* or *NRF2*, but also by epigenomic errors, exon skipping, and many other mechanisms in the genes encoding these regulatory proteins, which consequently make NRF2 stable.^14^^,^^15^^,^^16^^,^^17^^,^^18^^,^^19^^,^^20^ These activations of NRF2 lead to the hyperexpression of cytoprotective enzymes and also provoke metabolic reprogramming, conferring both malignant growth properties and resistance to anticancer radio/chemotherapy to cancer cells.^21^^,^^22^ The NRF2-hyperactivated cancers manifest strong resistance to currently standard therapies and bring about poor prognosis.

Multiple lines of clinical evidence suggest that the KEAP1-NRF2 pathway is an important pathway associated with resistance to ICB therapy.^23^^,^^24^^,^^25^ It has been reported that immune cell infiltration into tumor microenvironments serves as one of the critical signs of ICB resistance.^26^ Non-inflamed tumors or cold tumors are poorly infiltrated by lymphocytes.^27^ In addition, lower-level infiltrations of other types of immune cells are often observed in patients with KEAP1-mutated LUAD in the Cancer Genome Atlas (TCGA) database,^28^ the Tumor Immune Estimation Resource (TIMER) database,^29^ and the randomized phase III IMpower131 study.^30^^,^^31^ However, as *KEAP1* mutations frequently co-occur with *STK11* mutations in clinical samples, which is also known to modulate the tumor immune milieu,^23^^,^^32^^,^^33^ these clinical observations suggest that NRF2 may act collaboratively with STK11 to cause the lower-level immune cell infiltrations in the tumors. These observations lead us to hypothesize that NRF2 activation provokes the poor immune cell infiltrations. To examine whether NRF2 activation in cancer is the direct cause of reduced immune cell infiltration into tumors, several pioneer studies tried to generate mouse models of the NRF2-activated tumors. However, these mouse models recapitulate only partially the immunological phenotypes.^34^^,^^35^^,^^36^

In this study, therefore, we challenged the establishment of the 3LL syngeneic mouse model, which is the only reproducible syngeneic murine model for lung cancer.^37^ We generated 3LL lung cancer cell lines with NRF2 hyperactivation by means of the *Keap1* gene deletion and those with KEAP1-NRF2 concomitant deletion using the genome editing technology. These gene engineered cells were transplanted into C57BL/6 mice either unilaterally or bilaterally, and syngeneic tumors were successfully established. Analyses of these transplanted cancer tissues revealed that the immune cell infiltration is significantly decreased in the KEAP1-deleted/NRF2-activated tumors. CD45-positive cells including myeloid and monocytic cells, are mainly decreased, along with natural killer (NK) cells and dendritic cells (DCs). In contrast, the concomitant deletion of NRF2 reversed the poor immune cell infiltration in the KEAP1-deleted tumors. These results provide convincing lines of evidence that the NRF2 activation in cancer cells suppresses immune cell infiltration into tumors. Our observations shed light on the mechanistic basis of how NRF2-activation elicits cancer malignancy and contribute to the understanding of how we could develop NRF2-targeting therapies for NSCLC.

## Results

### Generation of NRF2-activated murine lung cancer cell line

It has been shown that NRF2 activated cancer cells reduce immune cell infiltration and affect the tumor microenvironment activity.^28^^,^^29^^,^^30^^,^^31^ However, model systems to elaborate the insights into influences of NRF2 addicted cancers toward tumor microenvironments have not been well established. To clarify functional impacts of the NRF2 activation in cancer cells on the formation and activity of tumor microenvironment *in vivo*, we decided to use the murine lung cancer cell line 3LL (Lewis lung carcinoma), as 3LL cells can form tumors upon transplanting into an immunocompetent mouse.^37^ 3LL is a lung cancer similar to human LUAD.^38^ It has been known that *KEAP1* somatic mutations occur more frequently in LUAD^39^ than lung squamous cell carcinoma. 3LL cells do not harbor somatic mutations in *Keap1* nor *Nrf2* genes.^38^

**Figure 1:**
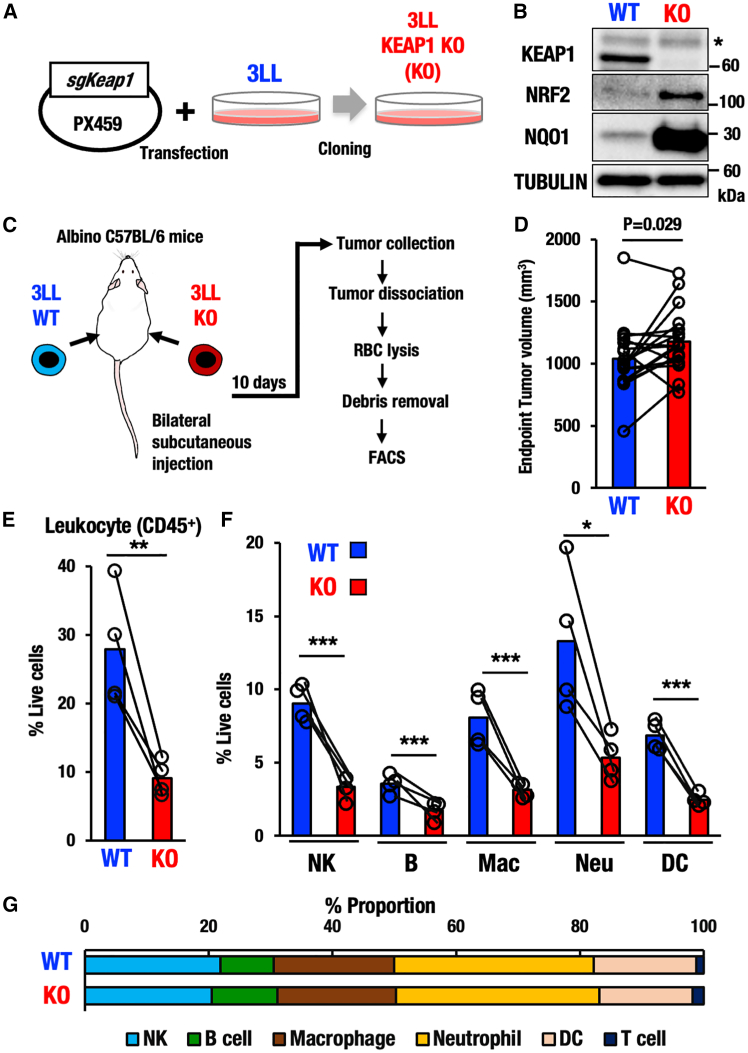
Decrease of immune cell infiltration in KEAP1-deleted tumors (A) Generation of NRF2-activated 3LL murine lung cancer cell line by deleting KEAP1. PX459 vector expressing *sgKeap1* was transfected into WT 3LL cancer cells. KEAP1-KO 3LL cancer cell lines were established by cloning from a single colony. (B) Western blot analysis of KEAP1, NRF2, and NQO1 in WT and KEAP1-KO 3LL cancer cell lines. Asterisk indicates a non-specific band. (C) Experimental scheme for the bilateral tumor transplantation model of WT and KEAP1-KO 3LL cells. After transplantations and tumor growth, tumors were taken, and flow cytometry analysis of the tumors was conducted as described. (D) Paired plot showing tumor volumes at endpoint in mice bilaterally injected with both WT and KEAP1-KO cancer cells. Each line connects tumor volumes from the same mouse (*n* = 14). (E) Percentages of CD45-positive leukocytes in live cells within the WT and KEAP1-KO tumors (*n* = 4, each). Note that CD45-positive cells were decreased significantly in KEAP1-KO cell-derived tumors. (F) Percentages of NK cell, B cell, macrophage (Mac), neutrophil (Neu), and DC in live cells within the WT and KEAP1-KO tumors (*n* = 4, each). (G) Proportional ratio of NK cell, B cell, macrophage, neutrophil, DC, and T cell in live cells within the WT and KEAP1-KO tumors. Note that the proportions of these cell types do not change much. Paired t-test. *p* < 0.05 was considered statistically significant; ∗*p* < 0.05, ∗∗*p* < 0.01, ∗∗∗*p* < 0.005. Data are presented as mean, and dots represent individual tumors.

We generated murine 3LL lung cancer cell lines with NRF2 activation by deleting the *Keap1* gene using the CRISPR-Cas9 genome editing method (Figure 1A). We have generated two cell lines with mutations in the *Keap1* gene. Sequencing analysis verified that all these KEAP1-KO (Knockout) 3LL cell lines harbored a homozygous 1 bp insertion in the *Keap1* gene exon 2, which corresponds to the BTB domain and caused a frameshift and led to the formation of a premature stop codon (Figure S1A). Immunoblot analysis confirmed that KEAP1 became undetectable in the KEAP1-KO cell line (Figure 1B). Conversely, NRF2 protein level was significantly increased along with that of *NAD(P)H:quinone oxidoreductase 1 (Nqo1)*, a representative NRF2 target gene in the KEAP1-KO cell line. To further validate the activation of NRF2, we examined transcript levels of *Nqo1* and *glutathione S-transferase alpha 4 (Gsta4)* genes and found significant upregulations of *Nqo1* and *Gsta4* mRNAs in the KEAP1-KO cell line (Figure S1B). Thus, we successfully generated 3LL cancer cell lines with marked NRF2 activation by means of CRISPR-Cas9-mediated mutations of the *Keap1* gene.

### NRF2-activated cancer cells provoke a severe immune cell infiltration decrease

To investigate the influences of the NRF2 activation in 3LL cancer cells on the tumor microenvironment, we challenged transplantation experiments of KEAP1-KO and wild type (WT) 3LL cell lines, taking advantage of the syngeneic immunocompetent nature of 3LL cells toward albino C57BL/6 mouse strain. We bilaterally transplanted the KEAP1-KO and WT cancer cells into right and left flanks of albino C57BL/6 mice, respectively, and compared the tumor immune microenvironment in the same host immune conditions (Figure 1C). Both KEAP1-KO and WT cancer cells were successfully engrafted into the albino C57BL/6 mice, and the KEAP1-KO tumors displayed moderately accelerated growth compared to WT tumors (Figure 1D), suggesting that the NRF2 activation in 3LL cells by the KEAP1 deletion promotes the cancer cell growth. We surmise that subtle changes in tumor growth may be attributable to the aggressive nature of the 3LL cells.

To examine the tumor immune microenvironment of WT and KEAP1-KO tumors, we collected tumor tissues and performed flow cytometry after tumor cell dissociation, red blood cell lysis, and debris removal (Figure 1C). Tumor-infiltrating immune cells such as macrophages and NK cells were identified by specific cell marker antibodies. Employed strategy is shown in Figure S2. It should be noted that cells positive for CD45 leukocyte common antigen were significantly decreased in the KEAP1-KO tumors compared with WT tumors (Figure 1E). While CD45^+^ leukocytes occupied approximately 30% of total cells within the WT 3LL engrafted tumors, the cell fraction reduced to less than 10% of total cells within the KEAP1-KO 3LL engrafted tumors. This result indicates that the infiltrations of wide-ranging CD45-positive immune cells are significantly reduced in the KEAP1-KO tumors.

We further assessed the composition of tumor infiltrating immune cells, assessing which types of immune cells are decreased or increased within the KEAP1-KO 3LL engrafted tumors. We found that the following five immune cell fractions, i.e., NK cells (CD45^+^NK1.1^+^), B cells (CD45^+^B220^+^), macrophages (CD11b^+^F4/80^+^), neutrophils (CD11b^+^Ly6G^+^), and DCs (CD11b^+^CD11c^+^MHCII^+^), were all significantly decreased in the KEAP1-KO tumors compared with those in the WT tumors (Figure 1F). While CD45-positive leukocytes were significantly decreased in the KEAP1-KO tumors compared with WT tumors, the proportional ratio of these five detected immune cells was comparable between the WT and KEAP1-KO tumors (Figure 1G). These results indicate that KEAP1 deletion and succeeding NRF2 activation cause a low level of immune infiltration in tumors without changing the proportion of five major immune cells.

### Decrease of immune cell infiltration into KEAP1-deleted tumors is unrelated to immunosuppressive cells

To examine whether the observed low-level immune cell infiltration in KEAP1-KO tumors is attributable to the enhancement of immunosuppressive immune cells, we next examined immunosuppressive cells such as myeloid-derived suppressor cells (MDSCs) and M2 macrophages.^40^^,^^41^ We found that the monocytic M-MDSCs (CD11b^+^Ly6C^high^Ly6G^–^) and polymorphonuclear (PMN)-MDSCs (CD11b^+^Ly6C^low^Ly6G^+^) were rather significantly decreased in the KEAP1-KO tumors (Figure S3). These results indicate that the low-level immune cell infiltration in KEAP1-KO tumors is unrelated to the increase of MDSCs, but numbers of MDSCs and their suppressor activity are rather decreased in the KEAP1-KO tumors.

We also examined immunosuppressive M2 macrophages (CD11b^+^F4/80^+^CD206^+^), and the number of M2 macrophages was comparable between WT and KEAP1-KO tumors (Figure S3). In addition, immunosuppressive regulatory T cells (T_reg_) were not detected in both WT and KEAP1-KO tumors. These results indicate that low-level immune cell infiltration into KEAP1-KO tumors is not attributable to the enhancement of immunosuppressive immune cells.

Additionally, we examined T cells in the tumors of WT and KEAP1-KO tumors. We found very small numbers of T cells, such as CD3^+^ cells and CD4^+^ cells, were infiltrated into both WT and KEAP1-KO tumors. Moreover, very low numbers of CD8^+^ cells were observed in both WT and KEAP1-KO tumors (Figure S3). These results are consistent with the previous observation that 3LL tumors display weak immunogenicity, and only low-level T-cell infiltration was observed in the 3LL-transplanted tumors.^42^^,^^43^ In addition to the low-level infiltration levels of T cell subpopulations, there were no substantial differences in the numbers of major T cell populations between the WT and KEAP1-KO tumors. We surmise that the low immunogenic nature of the 3LL tumor might result in these results. Taken together, the flow cytometry analyses of the immune cells in the 3LL transplanted tumors revealed that the infiltration of overall immune cells except for T cells into the tumor microenvironments was severely suppressed in NRF2-activated 3LL tumors due to KEAP1 deletion.

### Immunohistochemical analyses revealed a decrease in infiltrating immune cells in the stroma of KEAP1-deleted tumors

**Figure 2:**
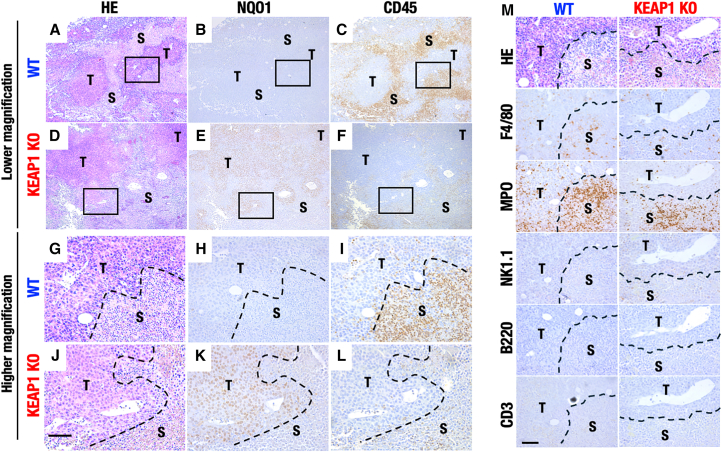
Immunohistochemical analyses of infiltrating immune cells in the stroma of KEAP1-deleted tumors (A–F) Representative images of HE-staining (A, D, G, and J), NQO1-immunostaining (B, E, H, and K), and CD45-immunostaining (C, F, I, and L) of WT (A–C and G–I) and KEAP1-KO (D–F and J–L) 3LL tumors. (G–L) Higher magnification images (G–L) were shown corresponding to the square area in lower magnification images (A–F). (M) Representative images of HE-staining and immunostaining for F4/80, MPO, NK1.1, B220, and CD3 in WT and KEAP1-KO tumors. T, tumor area; S, stroma area; Scale bars, 100 μm.

To validate the flow cytometry results showing that immune cell infiltration was reduced in the KEAP1-KO tumors, we carried out histological and immunohistochemical analysis of the tumors. As shown in Figures 2A and 2D, lower magnification images of Hematoxylin-Eosin (HE) stained tumors exhibited that tumor area (shown by T) and stroma area (shown by S) are observed similarly in both WT and KEAP1-KO tumor tissues. Higher magnification images showed that, while tumor cells have strong eosin staining with a larger nucleus, stroma cells exhibit faint eosin staining with a smaller nucleus (Figures 2G and 2J).

Immunohistochemical analysis revealed that NQO1 protein was expressed at a higher level in the tumor area of the KEAP1-KO tumor tissues (Figure 2E) than that of the WT tumor tissues (Figure 2B). Higher magnification images clearly showed that, while NQO1 expression in both tumor and stroma areas was very low in the WT tumor tissues (Figure 2H), a high level of NQO1 expression was detected in the tumor area but not in the stroma area of the KEAP1-KO tumor tissues (Figure 2K). These observations indicate that NRF2 is, in fact, activated in cancer cells of the KEAP1-KO tumor tissues.

By contrast to the NQO1 expression, immunohistochemical analysis of leukocyte common antigen CD45 revealed that CD45-positive immune cells resided at a higher level in the stroma of WT 3LL cancer tissues (Figure 2C), indicating massive infiltrations of immune cells to WT 3LL tumor tissues. Intriguingly, this CD45-positive immune cell infiltrations were severely abrogated in the stroma areas of KEAP1-KO tumor tissues (Figure 2F). Higher magnification images further clarified that, while many CD45-positive cells resided in the stroma areas of the WT tumor tissues (Figure 2I), much fewer CD45-positive cells were found in the stroma areas of the KEAP1-KO tumor tissues (Figure 2L). Further immunohistochemical analyses revealed that F4/80-positive macrophages and MPO-positive neutrophils were decreased in KEAP1-KO tumors compared with WT tumors, although their spatial distribution within the tumor tissue was not affected by the KEAP1 deletion (Figure 2M). NK cells and B cells were scarcely detectable in the tumors by immunohistochemical analyses, and T cells were also undetectable, in line with the flow cytometry findings showing minimal T cell populations in 3LL tumors. Taken together with the flow cytometric analyses, these results lead us to the hypothesis that immune cell infiltration into 3LL syngeneic tumor tissues is abrogated or heavily reduced in the KEAP1-KO tumors harboring high-level NRF2 activation.

### Unilateral transplantation reproduces reduced immune cell infiltration in the KEAP1-KO tumor

**Figure 3:**
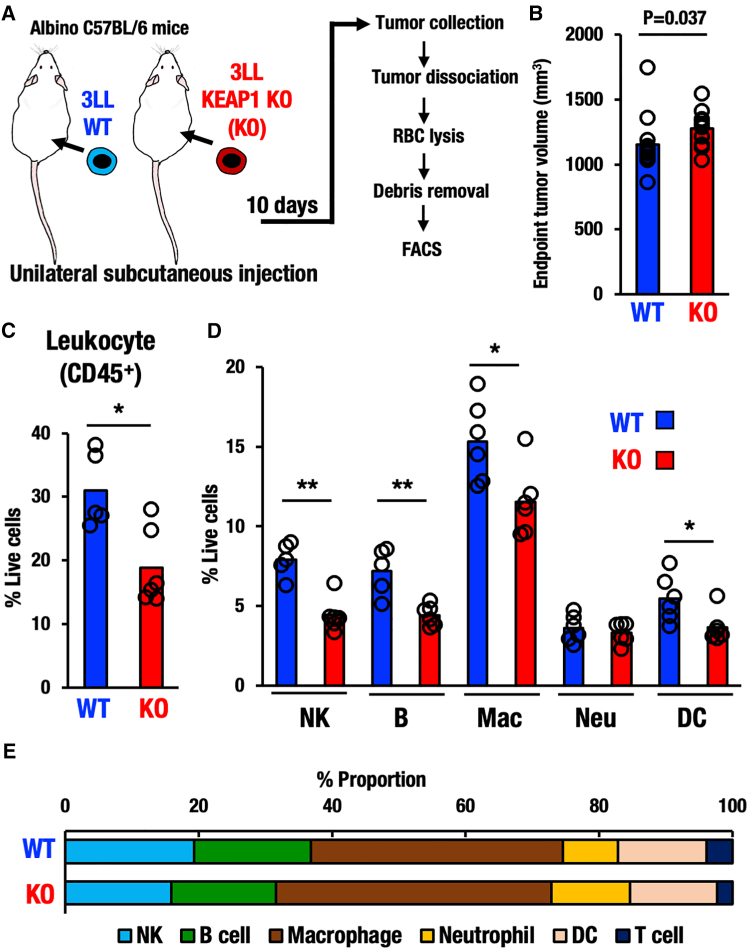
Unilateral tumor model showing that KEAP1 deletion suppresses the infiltration of immune cells (A) Experimental scheme for the unilateral transplantation tumor model of WT and KEAP1-KO 3LL cells and the flow cytometry protocols of tumor tissue. (B) Endpoint tumor volume of WT and KEAP1-KO tumors (*n* = 11–12). (C) Percentages of CD45-positive leukocytes that infiltrated into the WT and KEAP1-KO tumors (*n* = 6, each). (D) Percentage of NK cells, B cells, macrophages (Mac), neutrophils (Neu), and dendritic cells (DC) that infiltrated in the WT and KEAP1-KO tumors (*n* = 6, each). (E) Proportional ratio of NK cell, B cell, macrophage, neutrophil, DC, and T cell in live cells within the WT and KEAP1-KO tumors. Mann-Whitney U test. *p* < 0.05 was considered statistically significant; ∗*p* < 0.05, ∗∗*p* < 0.01, ∗∗∗*p* < 0.005. Data are presented as means, and dots represent individual tumors.

Thus far, we have examined immune cell infiltration by using bilateral transplantations of WT and KEAP1-KO 3LL cancer cells to albino C57BL/6 mice. While this procedure provides an elaborate comparison of immune cell infiltrations into WT and KEAP1-KO tumor tissues, there remains a possibility that both tumors residing bilaterally might affect the immune cell conditions mutually or might influence in a specific direction. Therefore, to eliminate the possibility that the injection of two different types of cancer cells into a single mouse bilaterally may provoke certain unpredictable effects on tumor microenvironments, we adopted a unilateral tumor model in which only one type of cancer cell was transplanted per mouse (Figure 3A).

KEAP1-KO tumors in the unilateral model exhibited comparable growth with WT tumors in individual mice (Figure 3B). Consistent with the finding in the bilateral model experiments, a significant reduction in CD45^+^ leukocytes was also observed reproducibly in KEAP1-KO tumors (19%) compared with those of WT tumors (31%) in the unilateral model (Figure 3C). It is important that the results of the unilateral transplantation model are, by and large, reproducible in the bilateral transplantation model. In both models, NRF2 activation in cancer cells suppresses the infiltration of immune cells into the tumor microenvironment.

Additionally, we noticed a couple of interesting observations in the comparison of unilateral and bilateral transplantation experiments. One is that the bilateral transplantation model displayed a much clearer difference in infiltrated CD45-positive leukocyte levels between WT and KEAP1-KO tumors (28% vs. 9%) compared with those of the unilateral transplantation model (31% vs. 19%). We surmise that as NRF2-activated cancer cells have an ability to suppress immune cell infiltration into tumors, circulating immune cells tend to accumulate more in WT 3LL tumors than in KEAP1-KO 3LL tumors in the bilateral model.

Another observation was made when we analyzed immune cell populations in the unilateral transplantation model and compared the results with those from the bilateral model. In the unilateral model, immune cells, including NK cells, B cells, macrophages, and dendritic cells, were all decreased in KEAP1-KO tumors compared to WT tumors. In contrast, the neutrophil level remained below 5% in both WT and KEAP1-KO tumors in the unilateral model (Figure 3D), whereas in the bilateral model, the neutrophil levels were 13% in WT tumors and 5% in KEAP1-KO tumors (Figure 1F). Additionally, macrophage levels differed between the two models: in the bilateral model, macrophage levels were 8% in WT tumors and 3% in KEAP1-KO tumors; in the unilateral model, they were 15% and 11%, respectively. These discrepancies may be attributed to differences in tumor numbers per mouse between the unilateral and bilateral transplantation experiments.

### NRF2 activation in cancer cells does not induce immune cells in systemic circulation

**Figure 4:**
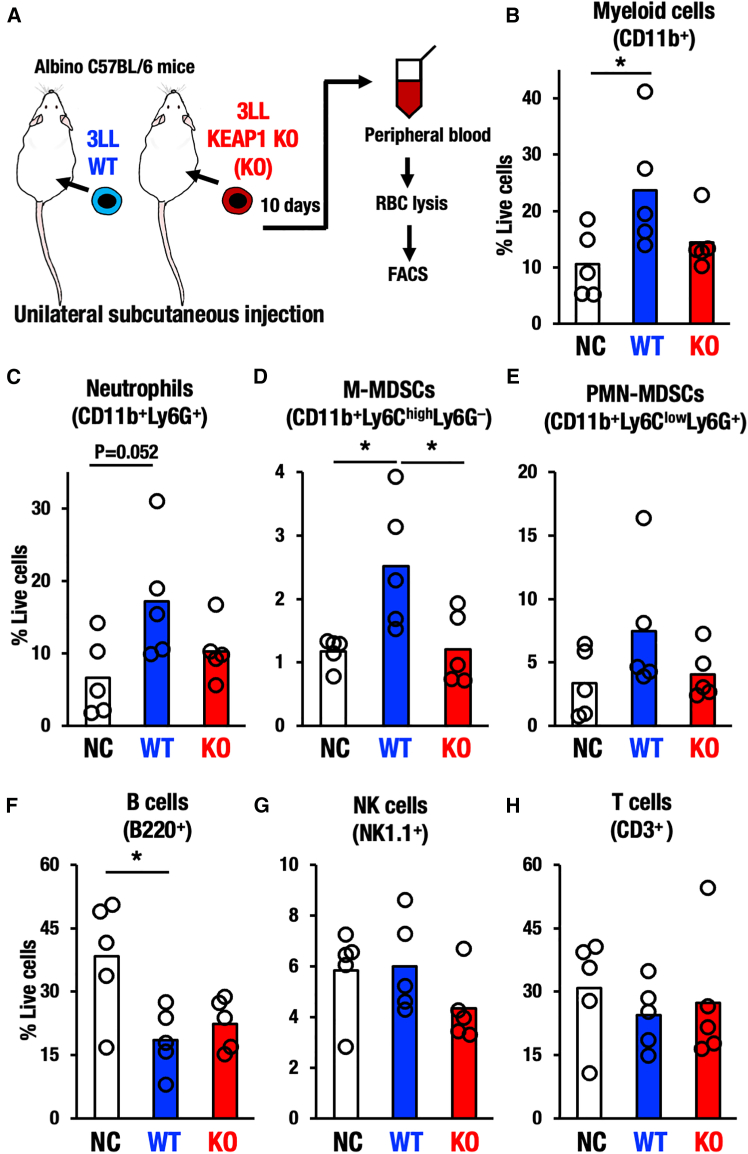
Immune cell populations in the circulation of the KEAP1-deleted tumor-bearing mice (A) Experimental scheme for the unilateral tumor transplantation model of WT and KEAP1-KO 3LL cell, and flow cytometry analysis of peripheral blood mononuclear cells from tumor-bearing mice and tumor non-transplanted control mice (NC). (B–H) Percentage of myeloid cells (B), neutrophils (C), M-MDSCs (D), PMN-MDSCs (E), B cells (F), NK cells (G), and T cells (H) in the peripheral blood from NC, WT, and KEAP1-KO tumor-bearing mice (*n* = 5, each). One-way ANOVA test followed by Tukey’s HSD test. *p* < 0.05 was considered statistically significant; ∗*p* < 0.05, ∗∗*p* < 0.01, ∗∗∗*p* < 0.005. Data are presented as mean, and dots represent individual animals.

Thus far, we have demonstrated that NRF2 activation reproducibly suppresses immune cell infiltration in tumors. These findings led us to hypothesize that the NRF2 activation in transplanted cancer cells might influence systemic immune conditions. To ask whether the transplantation of NRF2 activated tumor affects the immunological status of the whole body, we performed flow cytometry analysis to characterize immune cells in the peripheral blood of these tumor-bearing mice (Figure 4A). The employed strategy is shown in Figure S4. Significant increases of peripheral myeloid cells and neutrophils were observed in WT tumor-bearing mice compared with non-tumor-baring mice (Figures 4B and 4C). Consistent with this observation, it has been reported that cancer cells increase the number of circulating myeloid cells.^44^ It should be noted that peripheral myeloid cells and neutrophils in KEAP1-KO tumor-bearing mice were not increased (Figures 4B and 4C, respectively) despite the fact that the KEAP1-KO tumors show a comparable size with WT tumors. These results lead us to an important hypothesis that, while normal cancer cells induce immune cells in systemic circulation reflecting the anti-cancer inflammation, the NRF2-activated cancers acquire a unique ability to grow without inducing both immune cells in systemic circulation and anti-tumor inflammation.

To address this hypothesis, we examined immune suppressive cells, peripheral M-MDSCs, and PMN-MDSCs. While M-MDSC cells appeared to be increased in WT tumor-bearing mice compared with non-tumor-baring mice, the suppressive cells were not increased in KEAP1-KO tumor-bearing mice (Figure 4D). Peripheral PMN-MDSCs also showed similar trends to peripheral M-MDSCs (Figure 4E). These results were consistent with both MDSC levels in WT and KEAP1-KO tumor tissues (Figure S3). Thus, NRF2 activation in cancer cells reduces immune cell infiltration into tumors and the elevation of circulating immune cells in tumor-bearing mice by exploiting a distinct regulatory pathway from the MDSC pathways.

To address the influences of NRF2 activated cancer cells transplantation on the other lineages of systemic immunity, we also examined peripheral B cells, NK cells, and T cells. Number of peripheral B cells was decreased in both WT and KEAP1-KO tumor-bearing mice (Figure 4F), but the decrease was of a similar extent between WT and KEAP1-KO tumor-bearing mice, suggesting that the suppression of peripheral B cells in tumor-bearing mice is independent of the NRF2 activation in tumors. Peripheral NK cells and T cells were not significantly affected by tumor-bearing conditions (Figures 4G and 4H).

### Decrease of immune cell infiltration in KEAP1-KO tumors is attributable to NRF2 activation

To determine whether NRF2 activation is responsible for the decrease of immune cell infiltration in KEAP1-KO tumors, we decided to generate KEAP1 and NRF2 double knockout 3LL cells (DKO). To this end, we deleted *Nrf2* in the KEAP1-KO 3LL cells using the CRISPR-Cas9 genome editing method (Figure S5A). Sequencing analysis revealed that both 1 base insertion and 1 base deletion were heterozygously generated in exon 5, resulting in a premature stop codon by frameshift (Figure S5B). Immunoblot data of the KEAP1-NRF2-DKO 3LL cell line showed that both KEAP1 and NRF2 proteins were absent, and the NQO1 protein was markedly reduced (Figure S5C). The expressions of NRF2 target gene *Nqo1* and *Gsta4* were also downregulated in the DKO 3LL cell line, demonstrating successful reversal of NRF2 hyperactivation in the KEAP1-KO cancer cells (Figures S5D and S5E).

**Figure 5:**
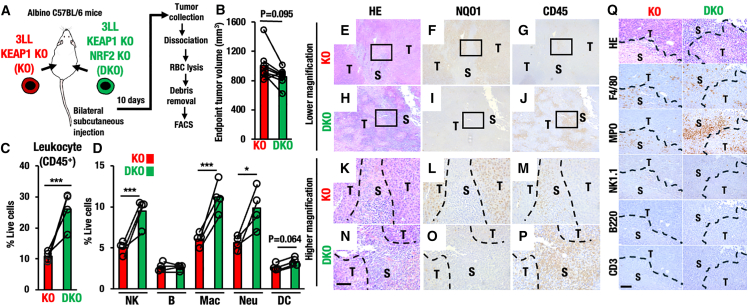
NRF2 deletion restores the KEAP1-deletion-mediated decrease of immune cell infiltration into tumors (A) Experimental scheme for the bilateral tumor transplantation model of KEAP1-KO and KEAP1-NRF2-DKO cells and the flow cytometry analysis of tumors. (B) Paired plot showing tumor volumes at endpoint in mice injected with both KEAP1-KO and KEAP1-NRF2-DKO cancer cells. Each line connects tumor volumes from the same mouse (*n* = 8). (C) Percentage of CD45-positive leukocytes in live cells within the KEAP1-KO and KEAP1-NRF2-DKO tumors (*n* = 4, each). (D) Percentages of NK cell, B cell, macrophage (Mac), neutrophil (Neu), and DC that infiltrated the KEAP1-KO tumors and KEAP1-NRF2-DKO tumors (*n* = 4, each). (E–P) Representative images of HE-staining (E, H, K, and N), NQO1-immunostaining (F, I, L, and O), and CD45-immunostaining (G, J, M, and P) of KEAP1-KO (E–G and K–M) and DKO (H–J and N–P) tumors. Higher magnification images (K–P) are shown corresponding to the square area in lower magnification images (E–J). (Q) Representative images of HE-staining and immunostaining for F4/80, MPO, NK1.1, B220, and CD3 in KEAP1-KO and DKO tumors. T, Tumor area; S, stroma area; Scale bars, 100 μm. Paired t-test. *p* < 0.05 was considered statistically significant; ∗*p* < 0.05, ∗∗*p* < 0.01, ∗∗∗*p* < 0.005. Data are presented as mean, and dots represent individual tumors.

Next, we bilaterally transplanted the KEAP1-KO and DKO cancer cells into both flanks of immunocompetent mice (Figure 5A). Concomitant deletion of NRF2 in KEAP1-KO tumors did not significantly change or moderately reduced tumor growth (Figure 5B). Importantly, while CD45^+^ immune cells in KEAP1-KO tumors were approximately 10% of total live cells, CD45^+^ immune cells were restored by approximately 25% in those of DKO tumors (Figure 5C), which was close to those in WT tumors of approximately 30%. These results provide compelling lines of evidence that NRF2 activation is responsible for the decrease of CD45^+^ immune cell infiltration in KEAP1-KO tumors. In a good agreement with the results of CD45^+^ immune cells, NK cells, macrophages, and neutrophils were restored after NRF2 deletion in KEAP1-KO tumors (Figure 5D).

By contrast, numbers of the B cells and DCs were comparable between the KEAP1-KO and DKO tumors (Figure 5D), suggesting that the decrease in B cells and DCs in the KEAP1-KO tumors is independent of the NRF2 hyperactivation. Consistent with the results, as B cells and DCs are not significantly changed between the KEAP1-KO and DKO tumors, there is an increased CD45^+^ immune cells in DKO tumors compared with KEAP1-KO tumors. Therefore, the proportional ratio of B cells and DCs appeared to be reduced (Figure S6). These results provide compelling lines of evidence that NRF2 activation by KEAP1 knockout is responsible for the reduction of NK cells and myeloid-lineage cells, including macrophages and neutrophils. However, the NRF2 knockout did not rescue the numbers of B cells and DCs.

To further ask whether low-level immune cell infiltration in the KEAP1-KO tumors is caused by NRF2 activation, we carried out histological and immunohistochemical analyses of the tumors. Lower and higher magnification images of HE staining of tumors showed that tumor area and stroma area are observed similarly in both KEAP1-KO (Figures 5E and 5K) and DKO tumor tissues (Figures 5H and 5N). Immunohistochemical analysis showed that high NQO1 expression in the tumor area of the KEAP1-KO tumor tissues (Figures 5F and 5L) was reversed in that of the DKO tumor tissues (Figures 5I and 5O). Higher magnification images clearly showed that high-level NQO1 expression was observed in tumor areas (shown by T) of the KEAP1-KO tumor tissues (Figure 5L), because of the strong NRF2 activation. By contrast, very low NQO1 expression was found in the tumor area of the DKO tumor tissues (Figure 5O), indicating that NRF2 activation in the KEAP1-KO tumor is efficiently canceled in the DKO tumor tissues.

Supporting the results of flow cytometry analyses, low and high magnification images of immunohistochemical analyses revealed that low-level CD45 expression was observed in the stroma area of the KEAP1-KO tumor tissues (Figures 5G and 5M), and CD45 was expressed at a high level in the stroma area of the DKO tumor tissues (Figures 5J and 5P). The immunohistochemical results revealed that F4/80-positive macrophages and MPO-positive neutrophils were increased in DKO tumors compared with KEAP1-KO tumors (Figure 5Q), consistent with the flow cytometry results. Thus, reduced immune cell infiltration due to NRF2 activation in KEAP1-KO tumors is efficiently rescued by the concomitant knockout of NRF2. We conclude based on these results that the NRF2 activation elicits the immune cell depleted conditions of KEAP1-KO tumors.

### Downregulation of immune system-related pathways in the NRF2-activated tumors

To our knowledge, this study experimentally demonstrates that NRF2 activation in cancer cells leads to reduced immune cell infiltration into tumors. To approach the mechanistic basis of how NRF2 activation induces the low-level immune cell infiltration into tumors, various experimental approaches can be applied to these experimental systems that we have developed, such as metabolome, transcriptome, and proteome analyses. As NRF2 acts as a transcription regulator, we decided to challenge RNA sequencing (RNA-seq) analysis, which provides a high-throughput and comprehensive analysis of gene expression, allowing us to capture global transcriptomic changes. We performed RNA-seq studies using WT, KEAP1-KO, and DKO tumor tissues.

**Figure 6:**
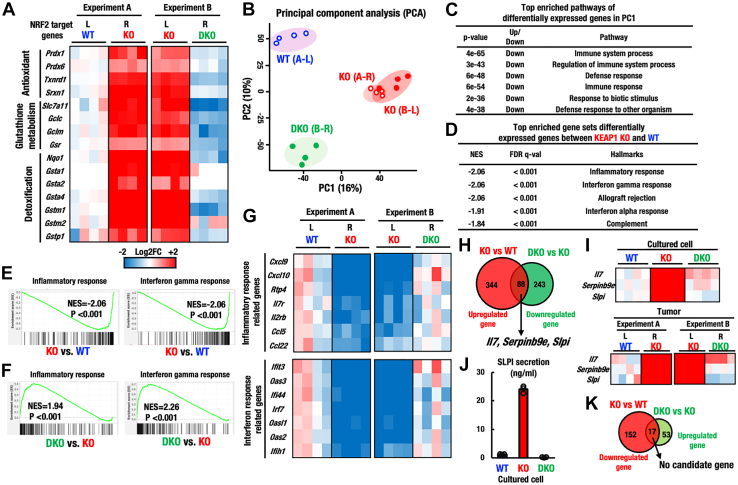
Downregulation of the immune system process pathway in KEAP1-deleted tumors (A) Heatmap shows the expression of representative NRF2-target genes in WT vs. KEAP1-KO tumors and KEAP1-KO vs. KEAP1-NRF2-DKO tumors (*n* = 4, each). Colors indicate the log2 fold change (FC) value relative to the mean expression level of WT tumors or KEAP1-KO tumors. (B) Principal component analysis (PCA) of RNA-seq data from WT vs. KEAP1-KO tumors, KEAP1-KO vs. KEAP1-NRF2-DKO tumors. The principal components, PC1 (16% variance explained) and PC2 (10% variance explained), are plotted. Each dot represents an individual sample. (C) Top enriched pathways of differentially expressed genes in PC1 of PCA analysis. (D) Top enriched gene sets of differentially expressed genes between KEAP1-KO and WT of GSEA analysis. (E and F) GSEA enrichment plot for gene sets inflammatory response and interferon gamma response pathway for KEAP1-KO vs. WT tumors (E), and KEAP1-NRF2-DKO vs. KEAP1-KO tumors (F). (G) Heatmap shows the expression of inflammatory response related genes and interferon response related genes in the WT vs. KEAP1-KO and KEAP1-KO vs. KEAP1-NRF2-DKO tumors (*n* = 4, each). Colors indicate the log2 FC value relative to the mean expression level of WT tumors. (H) Venn diagram showing the number of differentially expressed genes overlapped between KEAP1-KO upregulated (*p* < 0.01, log2FC > 2) and KEAP1-NRF2-DKO downregulated (*p* < 0.01, log2FC < −2) 3LL cancer cells. *Il7, Serpinb9*e, and *Slpi* genes are identified as candidate genes that may contribute to the low-level immune cell infiltration into tumors. (I) Heatmaps show the expression of *Il7*, *Serpinb9e* and *Slpi* genes in the WT, KEAP1-KO and KEAP1-NRF2-DKO 3LL cancer cells. Colors indicate the log2 FC values relative to the mean expression level of WT 3LL cancer cells. (J) SLPI secretion in the supernatant of cultured media from the WT, KEAP1-KO and KEAP1-NRF2-DKO 3LL cancer cells. Data are presented as mean, and dots represent individual samples. (K) Venn diagram shows the number of differentially expressed genes overlapped between KEAP1-KO downregulated (*p* < 0.01, log2FC < −2) and KEAP1-NRF2-DKO upregulated (*p* < 0.01, log2FC > 2) 3LL cancer cells. To our best knowledge, no candidate gene has been found that may contribute to reduced immune cell infiltration.

We examined the activation of the NRF2 pathway gene set in the WT and KEAP1-KO tumors, which were transplanted bilaterally into left and right flanks, respectively (Experiment A in Figure 6A). Heatmaps showed that expressions of many well-known NRF2 target genes related to antioxidant, glutathione metabolism, and detoxification were upregulated in KEAP1-KO tumors compared with those in WT tumors. We also validated the expressions of the same set of NRF2 target genes in the KEAP1-KO and DKO tumors that were transplanted into the left and right flanks, respectively (Experiment B). The high-level expressions of NRF2 target genes in KEAP1-KO tumors were mostly canceled in the DKO tumors (Figure 6A). These results nicely support our elaborate experimental settings in which NRF2 is activated in the KEAP1-KO tumors while suppressed in the DKO tumors.

To explore the wide associations between NRF2 activation and various unknown biological systems, which appear specifically in KEAP1-KO tumors, we performed principal component analyses (PCA) using the data obtained from comprehensive RNA-seq analysis (Figure 6B). We found that two independent datasets of KEAP1-KO (A-R and B-L from Experiment A and Experiment B, respectively) were almost indistinguishable, assuring the high reproducibility of the analyses. We found a stark separation of PC1 (principal component 1) between the WT and KEAP1-KO tumors. An important finding was a clear PC1 separation between the KEAP1-KO and DKO tumors. While WT and DKO tumors were not separated along PC1, they were separated along PC2, suggesting the influences of differentially expressed genes independent of NRF2. These results indicate that NRF2 activation provokes high-magnitude gene expression changes, which can be seen in the clear PC1 separation.

Intriguingly, top enriched pathways of differentially expressed genes in PC1 were mostly related to the immune responses, including both immune system process and regulation of immune system process (Figure 6C). These results suggest that the immune response is suppressed in KEAP1-KO tumors in an NRF2-dependent manner.

We next conducted a gene set enrichment analysis (GSEA) to explore differentially expressed gene sets in KEAP1-KO tumors. Consistent with the results of PCA and inferring expectation, top enriched gene sets differentially expressed between WT and KEAP1-KO tumors are inflammatory response and interferon gamma response (Figure 6D). GSEA enrichment plot comparing WT and KEAP1-KO tumors showed that genes related to these two sets were highly reduced in KEAP1-KO tumors (Figure 6E). We also conducted GSEA comparing KEAP1-KO and DKO tumors, and the enrichment plots showed that genes related to inflammatory response and interferon gamma response were highly enriched in DKO tumors (Figure 6F). These results support our conclusion that inflammatory response and interferon gamma response pathways are downregulated in KEAP1-KO tumors.

We hypothesized that the downregulation of inflammatory response and interferon gamma response pathways in KEAP1-KO tumors may be due to the low infiltration of immune cells into the tumors. To verify this hypothesis, we compared expression profiles of genes related to the inflammatory response and interferon gamma response pathways in KEAP1-KO tumors in comparison with those in WT tumors. To our expectation, the KEAP1-KO tumors showed significantly decreased expressions of genes related to these two pathways (Figure 6G). The suppressions of the inflammatory response and interferon gamma response pathway-related genes in KEAP1-KO tumors were almost completely restored in the DKO tumors. These results strongly support our contention that the infiltration of immune cells into the tumors is suppressed in KEAP1-KO tumors in an NRF2-dependent manner.

We also analyzed the expression of *Cd274* (PD-L1) and *Ctla4* mRNAs in the tumors using RNA-seq data (Figure S7). We found that, while *Cd279* (PD-1) expression was not detected in the tumors, both *Cd274* and *Ctla4* expression levels were reduced in KEAP1-KO tumors compared to WT tumors. This decrease was consistent with the reduced expression of T cell and myeloid cell markers, including *Cd4*, *Cd80*, and *Itgam* (Figure S7), suggesting that the downregulation of *Cd274* and *Ctla4* likely reflects decreased immune cell infiltration in KEAP1-KO tumors. By contrast, in DKO tumors, the reduced expression of *Cd274*, *Ctla4,* and immune cell markers was partially reversed, further supporting the hypothesis that NRF2 activation suppresses immune cell infiltration to the tumors.

### Expression of immunosuppressive factors in NRF2-activated cancer cells

To gain mechanistic insights as to how NRF2 activation in cancer cells induces low immune cell infiltration into tumor tissues, we investigated changes in gene expression in WT, KEAP1-KO, and DKO cancer cells. We found that, in comparison with WT cells, 344 genes were significantly upregulated in KEAP1-KO cells, whereas 243 genes were significantly downregulated in DKO cells (Figure 6H). Of these genes, 88 genes were concomitantly upregulated and downregulated in NRF2-activated KEAP1-KO cells and in NRF2-deleted DKO cells, respectively. Through the inspection of these 88 genes and several additional rounds of literature searches, we hypothesized that the following three genes, i.e., *Il7*, *Serpinb9e,* and *Slpi*, and their products, are plausible candidates. Of the three candidates, Interleukin-7 (IL-7) has been reported to promote the cell death of immune cells through continuous signaling,^45^ while Serine proteinase inhibitor 9 (SERPINB9) mediates tumor immune evasion by inhibiting apoptosis of cancer cells.^46^^,^^47^ Secretory leukocyte peptidase inhibitor (SLPI) has been reported to promote the tumorigenic and metastatic potential of cancer cells.^48^ Based on these lines of information, we surmise that these factors may contribute independently or in combination to the observed marked reduction of immune cell infiltration into NRF2 activated tumors.

To verify this hypothesis, we compared expressions of these genes in both cancer cells in culture and in transplanted tumors (Figure 6I). Heatmap of the RNA-seq data for the culture cells indicates that expressions of *Il7*, *Serpinb9e*, and *Slpi* genes were significantly upregulated in KEAP1-KO cancer cells, but were downregulated in DKO cancer cells (Figure 6I, left panel). Heatmap of the RNA-seq data for the tumors *in vivo* showed reproducible results that the expressions of *Il7*, *Serpinb9e,* and *Slpi* genes were upregulated and downregulated in KEAP1-KO tumors and DKO tumors, respectively (Figure 6I, right panel). These results evidently demonstrate that these candidate immune-suppressive factors are under the NRF2 regulation.

We further conducted ELISA experiments for IFN-γ, IL-7, and SLPI. IFN-γ and IL-7 were undetectable in the supernatant of all there 3LL cell lines: WT, KEAP1-KO, and DKO. In contrast, SLPI was markedly secreted by KEAP1-KO cells compared with WT and DKO cells (Figure 6J), indicating that SLPI is highly expressed and secreted in NRF2-activated 3LL cells. Given that SLPI has been implicated in immune suppression within the tumor microenvironment^49^^,^^50^ and systemic immune suppression,^51^^,^^52^ these findings support the notion that SLPI may contribute to the reduced immune cell infiltration observed in 3LL cells upon NRF2 activation.

We also found that, in comparison with WT cells, 152 genes were significantly downregulated in KEAP1-KO cells, whereas 53 genes were significantly upregulated in DKO cells (Figure 6K). Of these genes, 17 genes were concomitantly downregulated and upregulated in NRF2-activated KEAP1-KO cells and in NRF2-deleted DKO cells, respectively. We also conducted an inspection of these 17 genes and several additional rounds of literature searches, but to our best knowledge, no plausible candidate genes resided in this set of genes and their products.

## Discussion

**Figure 7:**
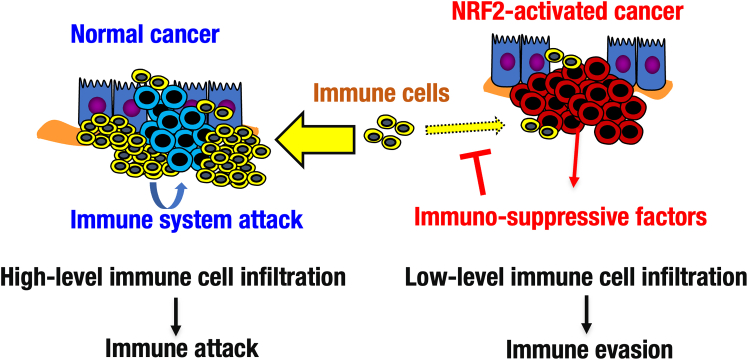
Proposed model for the NRF2-mediated suppression of immune cell infiltration in tumor A model showing that NRF2-activated cancer suppresses the infiltration of immune cells in tumors through up-regulating immune-suppressive factors.

Clinical observations suggest that NRF2 hyperactivation in cancer cells is associated with low-level immune cell infiltrations into tumors, it remains unclear how the NRF2 activation causes the reduction of immune cell infiltration. To examine this point experimentally, in this study, we established a mouse model of NRF2-activated cancers using the transplantation of syngeneic cells harboring *Keap1* and *Nrf2* gene modifications. As summarized in Figure 7, this study demonstrates that the NRF2 activation in cancer cells evokes low-level immune cell infiltration into tumors, which recapitulates the clinical phenotypes of the NRF2-activated cancers. Our mouse model revealed that the infiltration of immune cells is significantly decreased in the KEAP1-KO/NRF2-activated tumors, but concomitant knockout of NRF2 rescues the immunological phenotypes of the tumors. Transcriptome analyses suggest that three immune-suppressive genes under the NRF2 regulation may act either individually or in combination for the suppression of immune cell infiltration. Thus, our present cancer model study using syngeneic cancer cell transplantation unequivocally demonstrates that NRF2 hyperactivation in cancer cells provokes low-level immune cell infiltration to tumors or immune evasion through activating NRF2-dependent immune-suppressive genes, and this process contributes to the establishment of the malignancy of NRF2-activated tumors. Additionally, this study provides a valuable experimental model for understanding the mechanisms and developing therapies to combat the malignant NRF2-activated cancers.

### Validity of the mouse 3LL tumor transplantation model

The lower immune cell infiltration is reproducibly observed clinically in KEAP1-mutated LUAD cases, such as in the TCGA database,^28^ the TIMER database,^29^ and the IMpower131 study.^30^^,^^31^ Our mouse model study exhibits strong concordance with these available clinical data. Since the report showing that NRF2-activated cancer is resistant to ICB,^23^ many studies have been challenging the elucidation of underlying mechanisms. However, to the best of our knowledge, no mouse model that recapitulates these clinical phenotypes has been established. In this regard, the following observations may be pertinent. In the KEAP1-mutant *Kras*^*G12D/+*^*p53*^−/−^ lung cancers, the recruitment of CD103^+^ DCs was decreased, but CD45-positive cells were not influenced.^34^ M2-macrophages were increased in tumors generated by the orthologous transplantation of KEAP1 mutant LL2 cancer cells compared with those generated by WT LL2 cells.^35^ Myeloid cells were increased in tumors generated by the transplantation of KEAP1 mutant LKR10 cancer cells compared with those generated by WT LKR10 cells.^36^ While these pioneering studies provide important observations, the phenotypes observed do not reflect those typically observed in clinical cases. By contrast, this study established the mouse model that recapitulates the clinical phenotypes of low-level immune cell infiltration into NRF2-activated tumors using syngeneic 3LL cancer cells harboring genetic manipulations in *Keap1* and *Nrf2* genes.

### Mechanisms by how NRF2 activation in cancer cells induces the immune phenotype of tumors

Reduced expression of *MHC (Major histocompatibility complex)* genes was observed in NRF2-activated H460 cancer cells in culture.^53^ Indeed, our transplanted 3LL model also showed the reduced expression of *MHC* genes in the NRF2-activated KEAP1-KO tumors (Figure S8A). By contrast, in the case of cultured 3LL cancer cells, the expression of *MHC* genes was not significantly changed by the NRF2 activation (Figure S8B). The *MHC* genes, especially class II members, are normally expressed on antigen-presenting cells such as macrophages, DCs, and B cells.^54^ Therefore, we surmise that the decrease of *MHC* expression in our present *in vivo* transplantation data is attributable to the low-level immune cell infiltration into tumors, which reduces total expression levels of *MHC* genes in tumors *en bloc* without affecting the expression of *MHC* genes in NRF2-activated 3LL cancer cells. This interpretation is consistent with the results of flow cytometry and immunohistochemistry, showing that immune cell infiltration into the tumor microenvironment is suppressed in the NRF2-activated tumors.

In the search for responsible or pertinent genes for the decrease of immune cell infiltration, we identified three candidate genes, *Il7*, *Serpinb9e,* and *Slpi*, which are expressed in NRF2-activated cancer cells and may contribute to the induction of low-level immune cell infiltration into the NRF2-activated tumor. Intriguingly, we found NRF2 binding sites in the proximity to all three genes in the ChIP-seq data for human lung cancer cell lines^22^ and mouse bone marrow-derived macrophages.^55^ Especially, we found that SLPI is highly expressed and secreted in NRF2-activated 3LL cells, supporting the notion that SLPI may contribute to the reduced immune cell infiltration observed upon NRF2 activation in 3LL cells. Thus, it seems plausible that NRF2 directly activates the expression of these genes, and these gene products, either individually or in combination, repress the immune cell infiltration into tumors. Contributions of yet unidentified factors may also be possible.

An interesting observation in this study is that the transplantation of WT 3LL cells led to a clear increase in peripheral CD11b^+^ myeloid cells, suggesting a systemic pro-inflammatory and immune-activating effect. In contrast, such increases were attenuated in mice transplanted with NRF2-activated 3LL cells, consistent with our observations in the tumor microenvironment, where myeloid cell infiltration was also reduced. These findings suggest that NRF2 activation in tumor cells suppresses not only local immune cell recruitment within the tumor but may also attenuate the systemic inflammatory and immune response typically triggered by tumor-derived signals. This could be due to the NRF2-mediated suppression of pro-inflammatory cytokine and chemokine secretions from tumors, resulting in reduced mobilization and expansion of myeloid cells in both the tumor and peripheral compartments.

In conclusion, this study provides convincing lines of experimental evidence that the NRF2 activation in cancer cells suppresses immune cell infiltration into tumors, which recapitulates frequently observed clinical phenotypes of the NRF2-activated malignant cancers. Importantly, enfeebling the NRF2 activity can reverse the low-level immune infiltration phenotypes observed in NRF2-activated tumors. This study provides valuable insight into how NRF2-activated cancers acquire malignancy and further highlights the importance of developing NRF2-targeting therapies.

### Limitations of the study

In this study, we have evaluated immune cell infiltration in a syngeneic mouse model using the transplantation of 3LL lung cancer-derived cells with either NRF2 hyperactivation via *Keap1* gene deletion or concomitant *Keap1-Nrf2* gene deletion. This subcutaneous transplantation model clearly demonstrated that NRF2 activation represses immune cell infiltration. We anticipate that orthotopic transplantation models are also feasible and would provide additional relevant insights. We have conducted flow cytometric analyses, conventional immunohistochemistry, and RNA-seq analyses in this study. More precise characterization of immune cell repertoire within tumors can be achieved, although technically challenging, for example, by employing multiplex immunohistochemistry. In this study, we identified three candidate genes, *Il7*, *Serpinb9e,* and *Slpi*, are identified in this study, which appear to contribute to the induction of low-level immune cell infiltration into the NRF2-activated tumors. To substantiate the roles that these genes play, in-depth molecular biology and bioinformatic analyses would be highly informative.

## Resource availability

### Lead contact

Further information and requests for resources and reagents should be directed to and will be fulfilled by the lead contact, Masayuki Yamamoto (masayuki.yamamoto.c7@tohoku.ac.jp).

### Materials availability

All unique reagents generated in this study are available from the lead contact.

### Data and code availability

• The data of RNA-seq generated during this study have been deposited in GSE299332.
• This article does not report original code.
• Any additional information required to reanalyze the data reported in this article is available from the lead contact upon request.

## Acknowledgments

This work was supported in part by 10.13039/501100001700MEXT/10.13039/501100001691JSPS KAKENHI (19H05649, 21H05270 and 22K19450 to M.Y., and 22K06876 and 25K02449 to T.S.), the Research Support Project for Life Science and Drug Discovery (Basis for Supporting Innovative Drug Discovery and Life Science Research (BINDS)) from 10.13039/100009619AMED (JP23ama121038 to M.Y.), the Takeda Science Foundation, the Foundation of Promotion of Cancer Research, the Gonryo Medical Foundation, the Prince Takamatsu Cancer Research Fund, the 10.13039/100007434Suzuken Memorial Foundation, the 10.13039/501100007533Kobayashi Foundation for Cancer Research, and the 10.13039/501100008673Yasuda Medical Foundation (to T.S.). This work was supported by JST, the 10.13039/501100025020Establishment of University Fellowships towards the Creation of Science Technology Innovation, Grant Number JPMJFS2102. We thank Fumiki Katsuoka and Keiko Tateno for performing RNA-sequence analyses. We also thank Ikuo Hirano and the Biomedical Research Core of Tohoku University Graduate School of Medicine for technical support.

## Author contributions

H.W., T.S., and M.Y. designed the research and analyzed the data. H.W., A.Z., M.S., M.M., Yuka.T., and Yushi.T. conducted the experiments. H.W., T.S., and M.Y. wrote the article.

## Declaration of interests

The authors declare no competing interests.

## STAR★Methods

### Key resources table

**Table:** 

REAGENT or RESOURCE	SOURCE	IDENTIFIER
**Antibodies**		

Anti-CD3 for flow cytometry	Biolegend	Cat#100312; RRID:AB_312677
Anti-CD8 for flow cytometry	eBioscience	Cat#11-0081-85; RRID:AB_464916
Anti-CD4 for flow cytometry	BD Bioscience	Cat#561099; RRID:AB_394461
Anti-CD11b for flow cytometry	Biolegend	Cat#101236; RRID:AB_11203704
Anti-F4/80 for flow cytometry	Biolegend	Cat#123117; RRID:AB_893489
Anti-F4/80 for flow cytometry	Biolegend	Cat#123108; RRID:AB_893502
Anti-CD80 for flow cytometry	Biolegend	Cat#104713; RRID:AB_313134
Anti-CD11c for flow cytometry	Biolegend	Cat#117318; RRID:AB_493568
Anti-MHC II for flow cytometry	eBioscience	Cat#17-5321-81; RRID:AB_469454
Anti-CD45 for flow cytometry	BD Bioscience	Cat#559864; RRID:AB_398672
Anti-NK1.1 for flow cytometry	BD Bioscience	Cat#552878; RRID:AB_394507
Anti-B220 for flow cytometry	BD Bioscience	Cat#553088; RRID:AB_394618
Anti-CD206 for flow cytometry	Biolegend	Cat#141703; RRID:AB_10901166
Anti-Ly6G for flow cytometry	Biolegend	Cat#127613; RRID:AB_1877163
Anti-Ly6C for flow cytometry	BD Bioscience	Cat#553104; RRID:AB_394628
Anti-CD16/32 for flow cytometry	Biolegend	Cat#101320; RRID:AB_1574975
Anti-NQO1 for IHC/Western blotting	Abcam	Cat#ab2346; RRID:AB_302995
Anti-CD45 for IHC	Abcam	Cat#ab10558; RRID:AB_442810
Anti-CD3 for IHC	Abcam	Cat#ab16669; RRID:AB_443425
Anti-B220 for IHC	eBioscience	Cat#13-0452-81, RRID:AB_466448
Anti-Myeloperoxidase (MPO) for IHC	Abcam	Cat# ab208670, RRID:AB_2864724
Anti-NK1.1 for IHC	CST	Cat#39197, RRID:AB_2892989
Anti-F4/80 for IHC	Abcam	Cat#ab300421, RRID:AB_2936298
Anti-CD11b for IHC	Abcam	Cat#ab133357, RRID:AB_2650514
Biotin-conjugated goat anti-rabbit IgG	Proteintech	Cat#SA00004-2, RRID:AB_2890944
Anti-goat IgG/biotinylated	Dako	Cat#E0466; RRID:AB_3676677
Streptavidin/HRP	Dako	Cat#P0397
EnVision+ Dual Link System-HRP	Dako	Cat#K4061
Mouse SLPI DuoSet ELISA	R&D systems	Cat#DY1735-05
Mouse IFN-gamma Quantikine ELISA Kit	R&D systems	Cat#MIF00-1
Mouse IL-7 Quantikine ELISA Kit	R&D systems	Cat#M7000
Anti-KEAP1 for Western blotting	Watai et al.^56^	N/A
Anti-NRF2 for Western blotting	Cell Signaling Technology	Cat#D1Z9C; RRID:AB_2715528
Anti-α-TUBULIN	Sigma-Aldrich	Cat#T9026; RRID:AB_477593

**Chemicals, peptides, and recombinant proteins**		

Mildform 10N	FUJIFILM Wako	Cat#131-10317
Hydrogen peroxide	Nacalai Tesque	Cat#18411-25
Sodium citrate buffer (pH 6.0)	LSI Medience	Cat#RM102-C
Tris-Buffered Saline (TBS) Tablets, pH 7.6	Takara	Cat#T9141
RPMI 1640 medium	Nacalai	Cat#30264-56
Fetal bovine serum	Nichirei	Cat#S175012
Penicillin-streptomycin	Thermo	Cat#15140122

**Critical commercial assays**		

Lipofectamine 2000	Invitrogen	REF#11668-019
Sepasol-RNA I Super G	Nacalai Tesque	Cat#09379-97
ReverTra Ace qPCR RT Master Mix with gDNA Remover	Toyobo	Cat#FSQ-301
THUNDERBIRD Probe qPCR Mix	Toyobo	Cat#QPS-101
Tumor Dissociation Kit, mouse	Miltenyi Biotec	Cat#130-096-730
Debris Removal Solution	Miltenyi Biotec	Cat#130-109-398
RNA screen tape	Agilent	Cat#5067-5576
RNA screen tape Sample buffer	Agilent	Cat#5067-5577
MGIEasy rRNA Depletion Kit	MGI	Cat#1000005953
MGIEasy RNA Directional Library Prep Set	MGI	Cat#1000006386
DNBSEQ-G400RS High-Throughput Sequencing Set	MGI	Cat#1000016995
Protein Block Serum-Free	Dako	Cat#X0909
SignalStain DAB Substrate Kit	Cell Signaling Technology	Cat#8059S
Puromycin dihydrochloride	Invitrogen	Cat#A1113803

**Deposited data**		

RNA-seq data	This paper	GSE299332

**Experimental models: Cell lines**		

3LL cell line WT	Sato et al.^57^	NA
3LL cell line KEAP1-KO	This paper	NA
3LL cell line KEAP1-NRF2-DKO	This paper	NA

**Experimental models: Organisms/strains**		

Albino C57BL/6 male mice (B6N-Tyrc-Brd/BrdCrCrl)	The Jackson Laboratory	https://www.jax.or.jp/

**Oligonucleotides**		

See Table S1 for primers used for RT-qPCR	This paper	N/A
guide RNAs targeting exon 2 of *Keap1* gene F: 5′-TGTGTCCTGCACGTGATGAA-3’; R: 5′-TTCATCACGTGCAGGACACA-3′	This paper	N/A
guide RNAs targeting exon 5 of *Nrf2* gene F: 5′-TTACTCATCGATCTCCTCGC-3′ R: 5′-GCGAGGAGATCGATGAGTAA-3′	This paper	N/A

**Recombinant DNA**		

pSpCas9(BB)-2A-Puro (PX459) V2.0 vector	Addgene	#62988

**Software and algorithms**		

Galaxy platform	Jalili et al.^58^	https://usegalaxy.org
Salmon quant script (Galaxy)	Patro et al.^59^	https://usegalaxy.org
iDEP	Ge et al.^60^	http://bioinformatics.sdstate.edu/idep96/
GENCODE database	Frankish et al.^61^	https://www.gencodegenes.org
FACSVerse	BD Biosciences	https://www.bdbiosciences.com/
FlowJo software	BD Biosciences	https://www.flowjo.com/

### Experimental model and study participant details

#### Cells

The murine 3LL cell line^57^ was obtained from the Institute of Development, Aging and Cancer, Tohoku University and cultured in RPMI 1640 supplemented with 10% fetal bovine serum and 1% penicillin-streptomycin at 37°C in a humidified incubator with 5% CO_2_. The cells were routinely tested for mycoplasma contamination. To establish genetically modified 3LL cell lines, CRISPR-Cas9-mediated genome editing was performed.^62^ Single guide RNAs targeting exon 2 of *Keap1* (F:5′-TGTGTCCTGCACGTGATGAA-3’; R: 5′-TTCATCACGTGCAGGACACA-3′) and exon 5 of *Nrf2* (F:5′-TTACTCATCGATCTCCTCGC-3’; R: 5′-GCGAGGAGATCGATGAGTAA-3′) were cloned into the pSpCas9(BB)-2A-Puro (PX459) V2.0 vector. These constructs were transfected into 3LL cells using Lipofectamine 2000 (Invitrogen) following the manufacturer’s instructions. After 48 h, cells were treated with puromycin for 48 h to enrich successfully transfected cells for positive selection. The surviving cells were cultured for an additional 48 h before being plated at single-cell densities into individual wells of 96-well plates.

#### Animals

Suspensions of WT or KEAP KO 3LL cells (2×10^6^ cells in 100 μL PBS) were injected subcutaneously into the left or right flank of 6-week-old albino C57BL/6 male mice, which made visualization of tumors readily. Syngeneic transplantation was performed using only male mice, as the 3LL cell line was derived from male mice. Tumor growth was monitored by measuring size by calipers every two days. Tumor volumes were calculated using the formula: V (mm^3^) = L × W^2^/2, where L is tumor length and W is tumor width. The allografts were allowed to grow for 10 days, after which the tumors were removed and rapidly frozen in liquid nitrogen. Mice were maintained according to the regulations of The Standards for Human Care and Use of Laboratory Animals of Tohoku University. All animal experiments were executed with the approval of the Tohoku University Animal Care Committee (ethical committee reference number 2024#001 and 2024#009).

### Method details

#### Western blotting

Whole-cell extracts were prepared in a sample buffer containing 20% glycerol, 4% SDS, 0.1-M Tris-HCl [pH 6.8], 12% 2-mercaptoethanol and bromophenol blue. After heat denaturation, the protein samples were subjected to SDS-polyacrylamide gel electrophoresis (SDS-PAGE) and electro-transferred to PVDF membranes. Specific protein signals were detected by anti-KEAP1^56^ (1:200 dilution), anti-NRF2 (D1Z9C, Cell Signaling Technology; 1:1000 dilution), anti-NQO1 (ab2346, Abcam; 1:1000 dilution) or anti-α-TUBULIN (T9026, Sigma-Aldrich; 1:1000 dilution) and the corresponding secondary antibodies.

#### Gene expression analysis

Total RNA was extracted from cancer cells *in culture* or tumor tissues *in vivo* using Sepasol-RNA I Super G (09379-97; Nacalai Tesque). The RNA concentration was measured using a NanoPhotometer NP80 (Implen). RNA was reverse transcribed into cDNAs using ReverTra Ace qPCR RT Master Mix with gDNA Remover (FSQ-301; Toyobo) according to the manufacturer’s instructions. The resulting cDNA was used as a template for quantitative reverse transcription PCR (RT-qPCR) using a THUNDERBIRD Probe qPCR Mix (QPS-101; Toyobo) with a QuantStudio 6 Flex (Life Technologies).^63^ The *Hprt* gene was used as an internal control. The primer and probe sequence for RT-qPCR are listed in Table S1.

#### Flow cytometry

Tumors were harvested from mice transplanted with WT 3LL cells, KEAP1-KO 3LL cells or DKO 3LL cells. Tumor cells were isolated using Tumor Dissociation Kit mouse (Miltenyi Biotec), following the protocol provided by the manufacturer. Red blood cells (RBC) were removed using RBC Lysis buffer (0.015 M NH_4_Cl, 1 mM KHCO_3_ and 10 μM EDTA-2Na). Debris was removed using Debris removal solution (Miltenyi Biotec). Propidium iodide (1 μg/mL) was used to remove dead cells. Analyses and cell sorting were performed using FACSVerse (BD Biosciences). Data were analyzed using FlowJo software (BD Biosciences). Antibodies used for flow cytometric analyses are described in STAR Methods key resources table.

#### RNA-sequence analysis

Total RNA was extracted from cancer cells or tumor tissues using the Sepasol-RNA I Super G. RNA quantity and purity were assessed using NanoPhotometer NP80 (Implen). RNA integrity (RIN) was assessed using TapeStation (Agilent). rRNA fraction was removed using an MGIEasy rRNA depletion kit (1000005953; MGI). Double-stranded DNA (dsDNA) libraries were created from the rRNA-depleted eluate using an MGIEasy RNA directional library prep kit (1000006386; MGI). The libraries were sequenced on a DNBSEQ-G400RS (MGI) system with a DNBSEQ-G400RS high-throughput sequencing kit V1.0 (1000016995; MGI) to obtain 150 paired-end reads. The sequencing data were processed on the Galaxy server (https://usegalaxy.org).^58^ Transcript per million (TPM) normalized read counts were generated using Salmon quant script (Galaxy version 1.5.1+galaxy0)^59^ with mouse reference transcript sequences (version 35) downloaded from GENCODE database (https://www.gencodegenes.org).^61^ The PCA and differential gene expression analysis were conducted on the iDEP platform (http://bioinformatics.sdstate.edu/idep/).^60^

#### Histological analysis

For HE staining, tumor tissues were fixed with Mildform 10N (131–10317; FUJIFILM Wako). The fixed tissues were embedded in paraffin, sliced into 4-μm-thick sections, and stained with HE. For immunohistochemistry, paraffin sections were rehydrated, autoclaved in 10 mmol/L sodium citrate buffer (pH 6.0) for antigen retrieval, treated with 3% H_2_O_2_, blocked with Protein Block Serum-free (X0909; Dako), and sequentially incubated with primary antibodies using anti-NQO1 (ab2346, Abcam; 1:500 dilution), anti-CD45 (ab10558, Abcam; 1:1000 dilution), anti-CD3 (ab16669, Abcam; 1:100 dilution), anti-MPO (ab208670, Abcam; 1:1,000 dilution), anti-F4/80 (ab300421, Abcam; 1/5,000), anti-CD11b (ab133357, Abcam; 1/4,000) and anti-B220 (13-0452-81, eBioscience; 1:400 dilution) for 16 h at 4°C. For NK1.1 immunohistochemistry, paraffin sections were rehydrated, autoclaved in 1 mM EDTA-2Na, 10 mM Tris-HCl buffer (pH 9.0) for antigen retrieval, treated with 3% H_2_O_2_, blocked with Protein Block Serum-free (X0909; Dako), and sequentially incubated with primary antibodies using anti-NK1.1/CD161 (39197, Cell Signaling Technology; 1:300 dilution) for 16 h at 4°C. For unconjugated primary antibodies, the corresponding secondary antibodies were used.

#### ELISA

WT 3LL cells, KEAP1-KO 3LL cells or DKO 3LL cells (1 x 10^5^ cells/mL) were cultured for 2 days in a 6-well plate using RPMI medium plus 10% fetal bovine serum. An aliquot of the cell culture supernatant was removed and assayed for measurement of interferon gamma (MIF00; R&D), IL-7 (M7000; R&D) and SLPI (DY1735-05; R&D) following the manufacturer’s procedures.

### Quantification and statistical analysis

Statistical significance was evaluated by Paired t-test, Mann-Whitney U test, or One-Way ANOVA followed by Tukey’s HSD test. *p* < 0.05 was considered statistically significant; ∗*p* < 0.05, ∗∗*p* < 0.01, ∗∗∗*p* < 0.005.
