# Supplementary material for: NRF2 activation in cancer cells suppresses immune infiltration into the tumor microenvironment
Source: iScience. 2025 Sep 6;28(10):113519. doi: 10.1016/j.isci.2025.113519 (PMC12483591; doi:10.1016/j.isci.2025.113519)
Supplement: Document S1. Figures S1–S8 and Table S1 [file mmc1.pdf]

iScience, Volume *28*

Supplemental information

NRF2 activation in cancer cells suppresses immune infiltration into the tumor microenvironment

Huaichun Wen, Takafumi Suzuki, Anqi Zhang, Miu Sato, Mahiro Matsumoto, Yuka Takahashi, Yushi Takahashi, and Masayuki Yamamoto

Supplementary Figure Legends

Supplementary Figure 1. Generation of KEAP1-deleted 3LL cell lines. **(A)** Sequencing analysis of KEAP1-KO 3LL cancer cell line. CGG (shown in blue) and underline indicate PAM (protospacer adjacent motif) sequence and guide RNA sequence, respectively. Note that 1 bp-insertion resulted in frameshift and premature stop codon. **(B)** Relative mRNA levels of the *Nqo1* and *Gsta4* in WT and KEAP1-KO 3LL cancer cell line (n=4). Mann-Whitney U test. $P<0.05$ was considered statistically significant; *$P<0.05$, **$P<0.01$, ***$P<0.005$. Data are presented as mean, and dots represent individual samples.

Supplementary Figure 2. Gating strategy of flow cytometry analyses to identify leukocytes, NK cells, B cells, myeloid cells, macrophages, neutrophils and DCs in tumor tissues. After removing debris, live singlet cells were used for analysis.

Supplementary Figure 3. Percentage of M-MDSCs, PMN-MDSCs, M2 macrophages, T_{reg}, $CD3^+$T cells, $CD4^+$ T cells and $CD8^+$ T cells that infiltrated in the WT and KEAP1-KO tumors (n=4, each). Paired t-test. $P<0.05$ was considered statistically significant; *$P<0.05$, **$P<0.01$, ***$P<0.005$. N.D. not detected. Data are presented as mean, and dots represent individual tumors.

Supplementary Figure 4. Gating strategy of flow cytometry analyses to identify myeloid cells, M-MDSCs, PMN-MDSCs, neutrophils, B cells, NK cells and $CD3^+$T cells in the peripheral blood from NC, WT and KEAP1-KO tumor-bearing mice.

Supplementary Figure 5. Generation of KEAP1-NRF2-DKO 3LL cell lines. **(A)** Scheme for generation of KEAP1-NRF2-DKO 3LL cell lines. PX459 vector expressing *sgNrf2* was transfected to KEAP1-KO 3LL cancer cells. KEAP1-NRF2-DKO 3LL cancer cell lines were established by cloning from a single colony. **(B)** Sequencing analysis of *Nrf2* gene locus in KEAP1-NRF2-DKO 3LL cell line. TGG (shown in blue) and underline indicate PAM sequence

and guide RNA sequence, respectively. **(C)** Western blot analysis of KEAP1, NRF2 and NQO1 in WT, KEAP1-KO and KEAP1-NRF2-DKO 3LL -cell line. Asterisk indicates non-specific band. **(D, E)** Relative mRNA levels of the *Nqo1* (**D**) and *Gsta4* (**E**) in WT, KEAP1-KO and KEAP1-NRF2-DKO 3LL cell line (n=4). One-way ANOVA test followed by Tukey's HSD test. $P<0.05$ was considered statistically significant; *$P<0.05$, **$P<0.01$, ***$P<0.005$. Data are presented as mean, and dots represent individual samples.

Supplementary Figure 6. Proportional ratios of NK cell, B cell, macrophage, neutrophil, DC and T cell in live cells within the KEAP1-KO and KEAP1-NRF2-DKO tumors.

Supplementary Figure 7. Heatmap shows expression of genes encoding immune cell markers in the WT, KEAP1-KO and KEAP1-NRF2-DKO tumors. Colors indicate the log2 FC value relative to the mean expression level of WT 3LL tumors.

Supplementary Figure 8. Expression of genes encoding MHC molecules in the WT, KEAP1-KO and KEAP1-NRF2-DKO tumors and cultured 3LL cells. (**A**) Heatmap shows expression of genes encoding MHC molecules in the WT, KEAP1-KO and KEAP1-NRF2-DKO tumors. Colors indicate the log2 FC value relative to the mean expression level of WT 3LL tumors. (**B**) Heatmap shows expression of genes encoding MHC molecules in the WT, KEAP1-KO and KEAP1-NRF2-DKO 3LL cancer cells. Colors indicate the log2 FC value relative to the mean expression level of WT 3LL cancer cells.

Supplementary Figure 1 (Related to Figure 1)

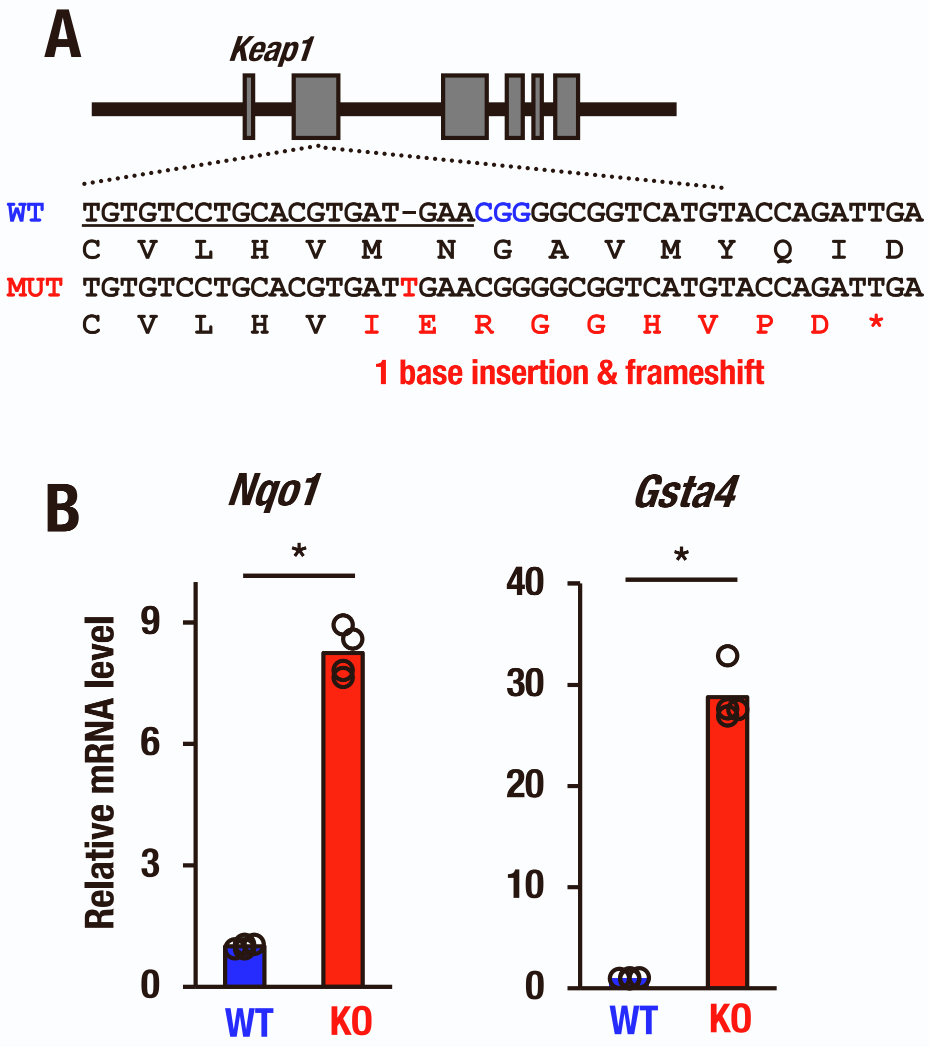

Supplementary Figure 2 (Related to Figure 1)

Tumor gating strategy

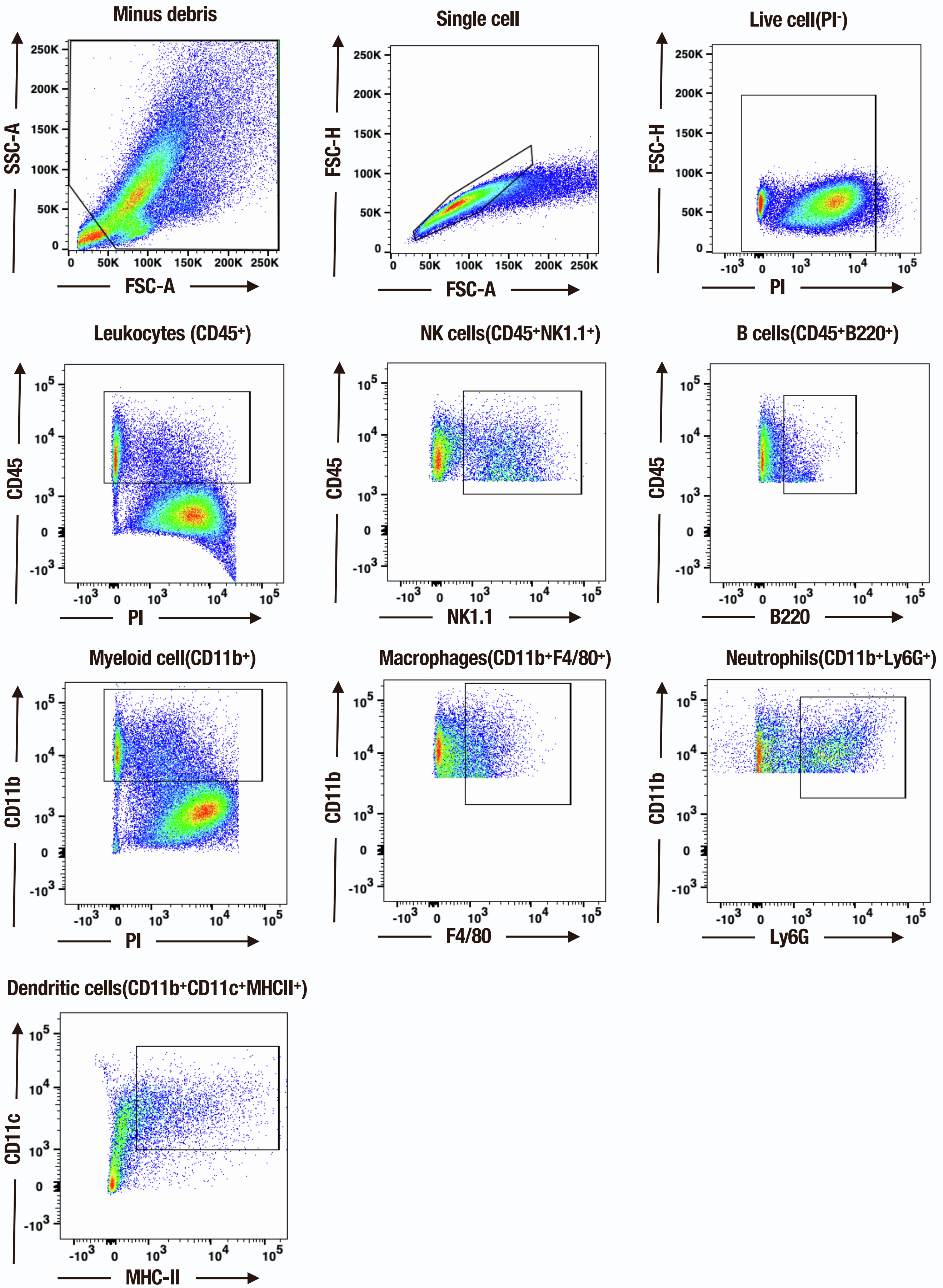

Supplementary Figure 3 (Related to Figure 1)

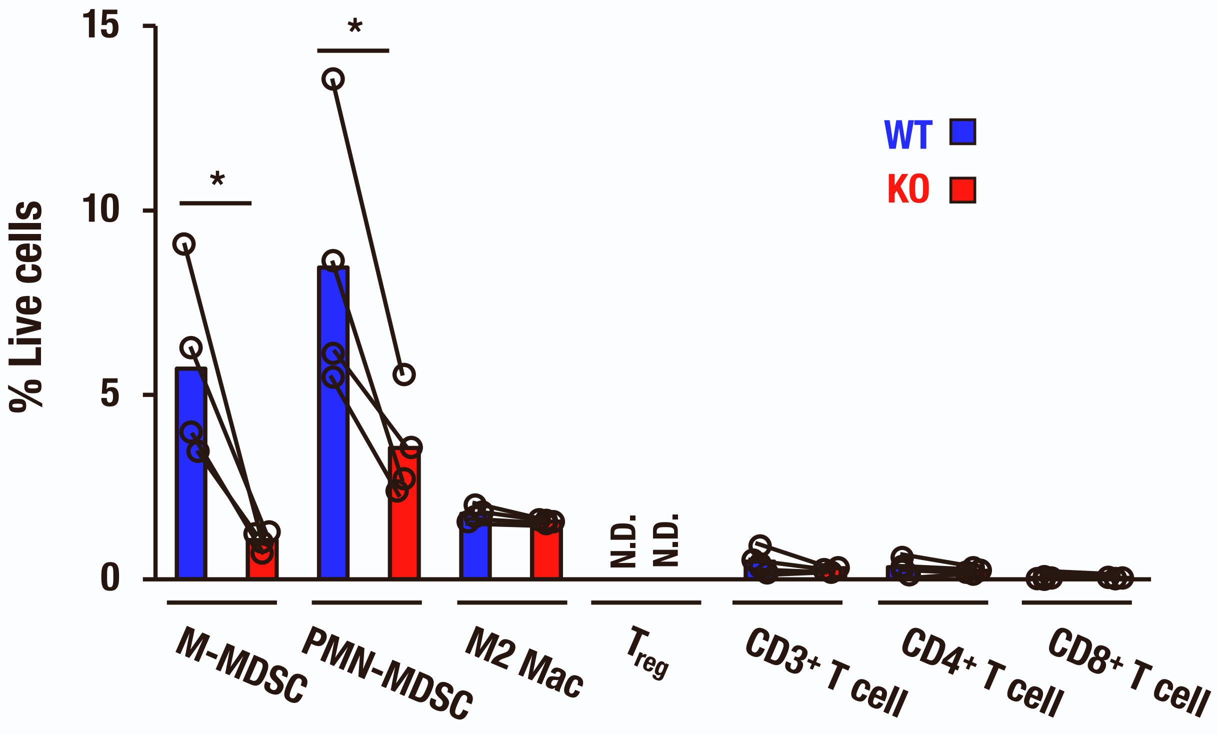

Supplementary Figure 4 (Related to Figure 4)

Blood gating strategy

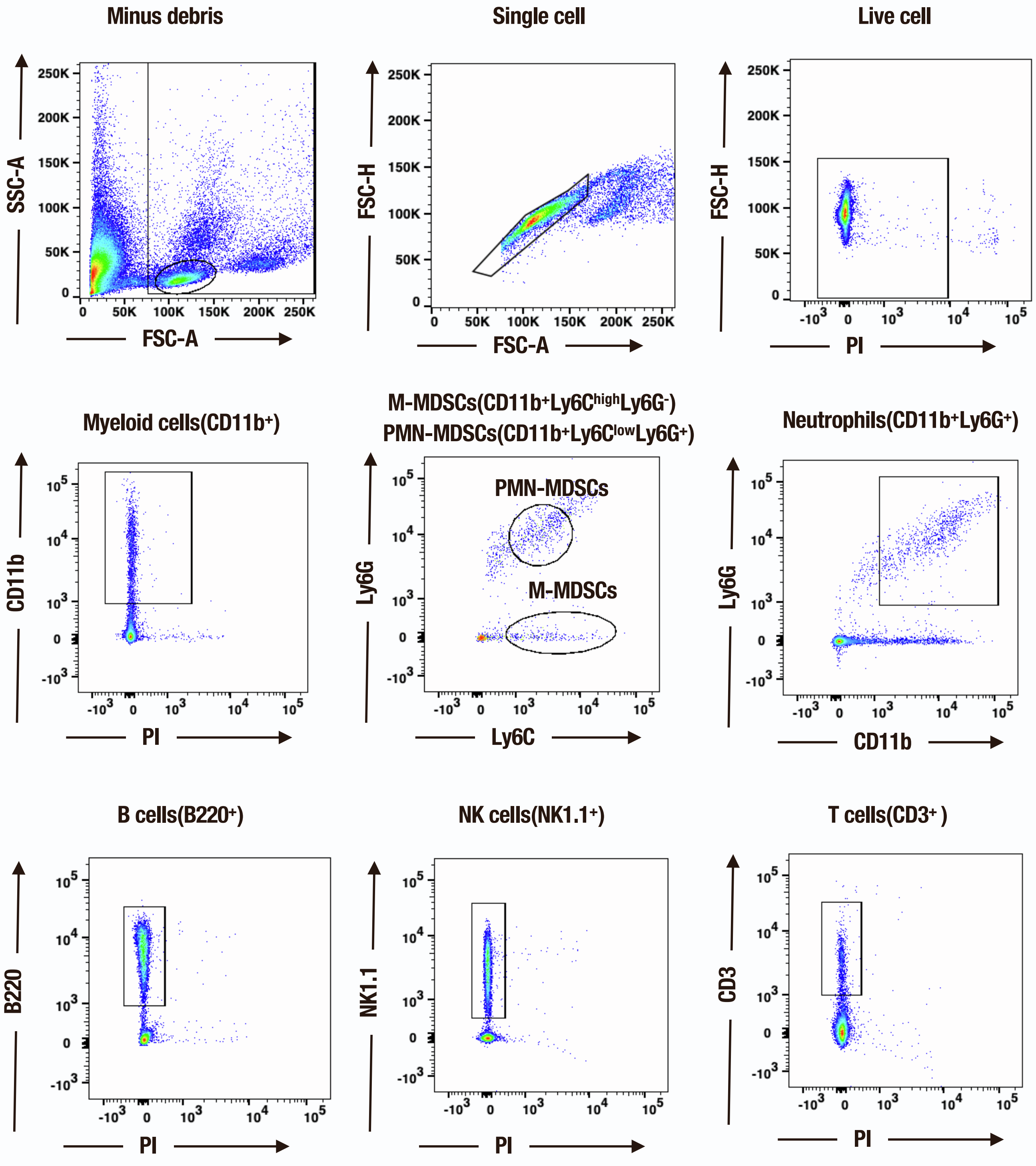

Supplementary Figure 5 (Related to Figure 5)

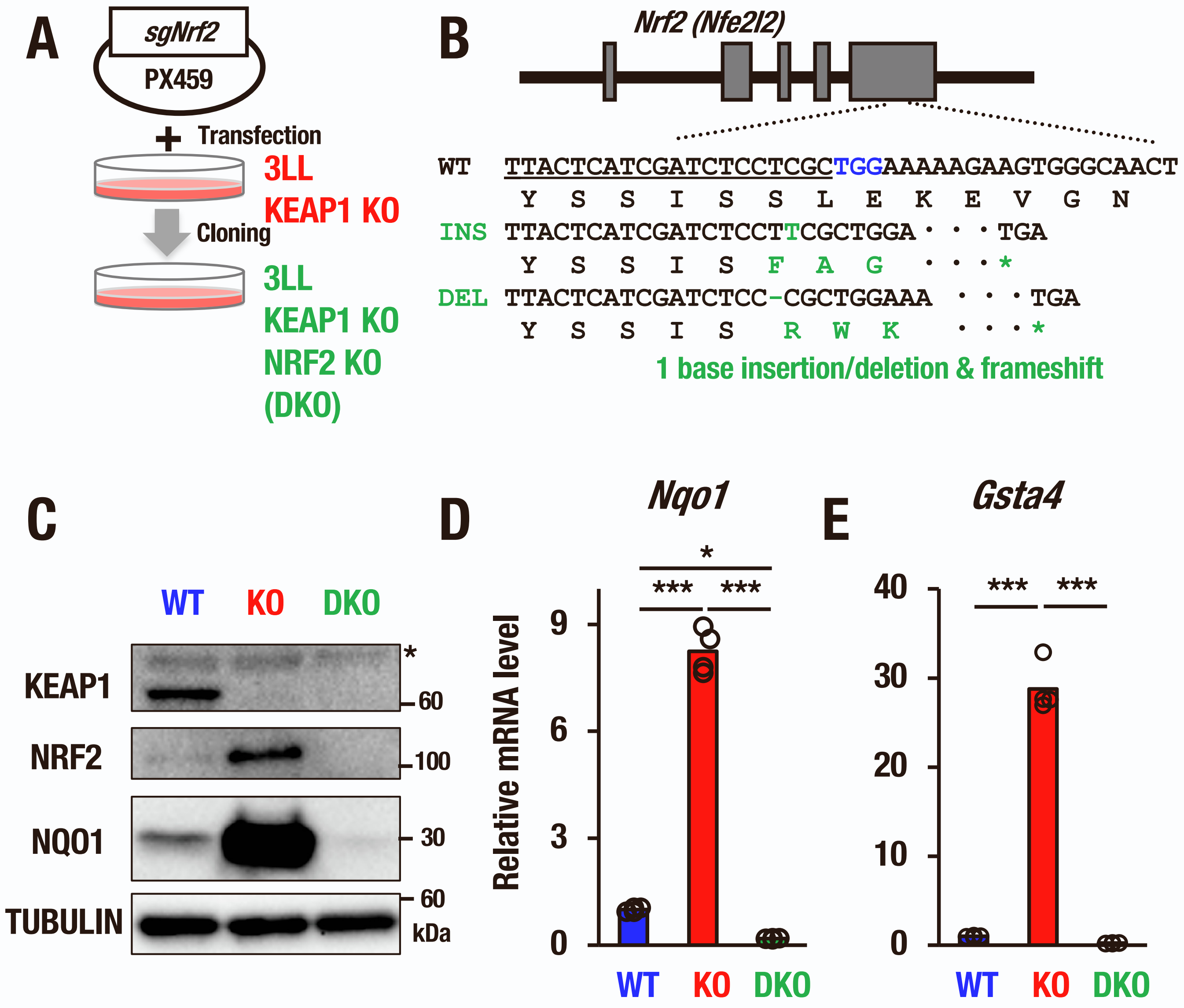

Supplementary Figure 6 (Related to Figure 5)

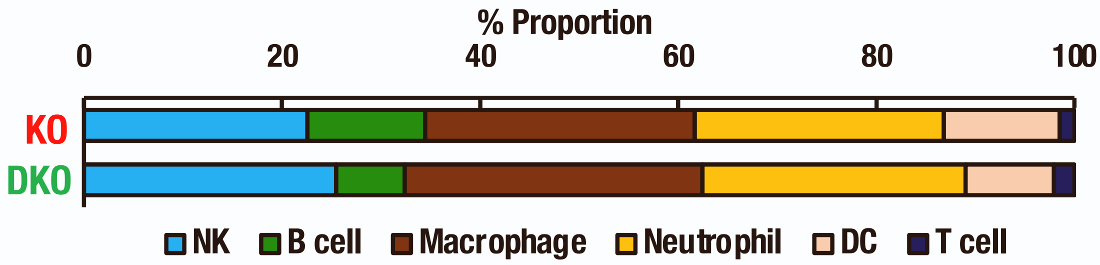

Supplementary Figure 7 (Related to Figure 6)

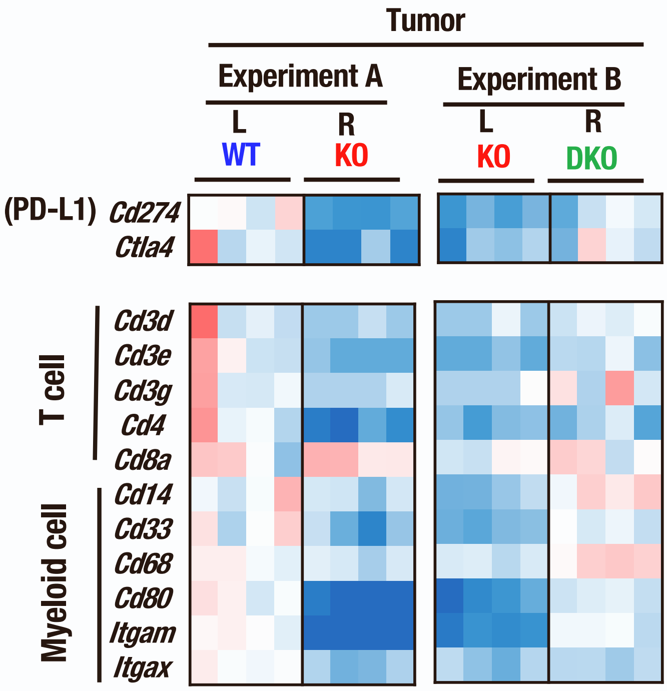

Supplementary Figure 8 (Related to Figure 6)

A

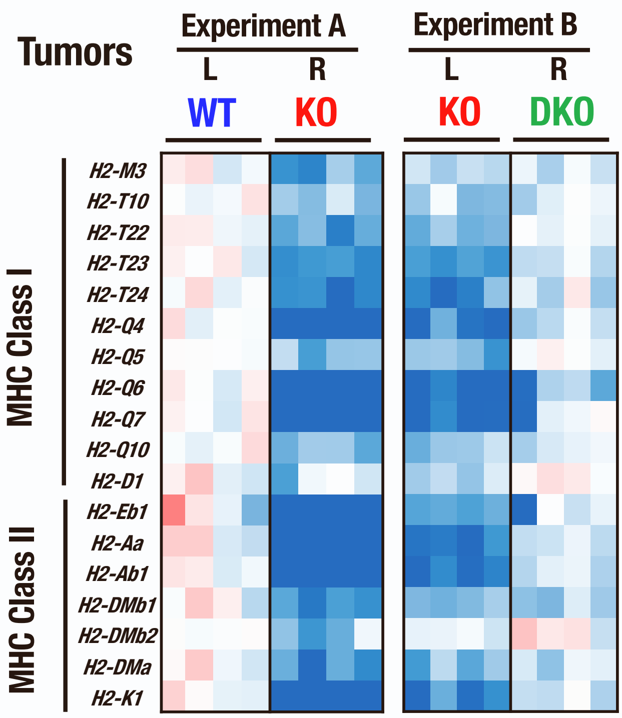

B

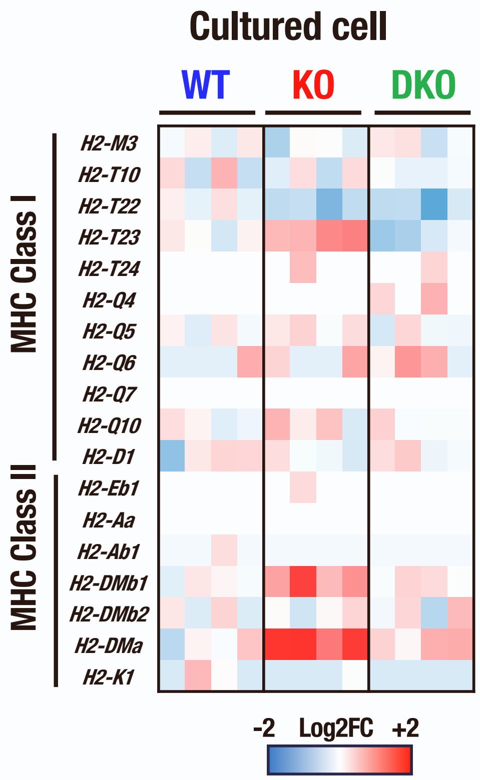

Supplementary Table 1

Primers and probes used for RT-qPCR

Gene	Sequence
HPRT-F	CTGGTGAAAAGGACCTCTCG
HPRT-R	TGAAGTACTCATTATAGTCAAGGG
HPRT-P	ATCCAACAAAGTCTGGCCTGTATCCAAC
NQO1-F	AGCTGGAAGCTGCAGACCTG
NQO1-R	CCTTTCAGAATGGCTGGCA
NQO1-P	ATTTCAGTTCCCATTGCAGTGGTTTGGG
GSTA4-F	GGGAACAGTATGAGAAGAAGATGCAAAA
GSTA4-R	CCCATCGATTTCAACCAAGG
GSTA4-P	TGCACACCTGCTTTTCGGCCAAG
